# Temperature Investigation on 3C-SiC Homo-Epitaxy on Four-Inch Wafers

**DOI:** 10.3390/ma12203293

**Published:** 2019-10-10

**Authors:** Ruggero Anzalone, Massimo Zimbone, Cristiano Calabretta, Marco Mauceri, Alessandra Alberti, Riccardo Reitano, Francesco La Via

**Affiliations:** 1 STMicroelectronics, Stradale Primosole, 50, 95121 Catania, Italy; 2 IMM-CNR, VIII Strada, 5, 95121 Catania, Italy; massimo.zimbone@imm.cnr.it (M.Z.); cristiano.calabretta@imm.cnr.it (C.C.); alessandra.alberti@imm.cnr.it (A.A.); 3 LPE, XVI Strada, 95121 Catania, Italy; Marco.Mauceri@lpe-epi.com; 4 Department of Physics and Astronomy, Via S. Sofia 64, 95100 Catania, Italy; riccardo.reitano@ct.infn.it

**Keywords:** 3C-SiC homo-epitaxy, CVD, bulk growth, growth temperature, KOH, stacking faults

## Abstract

In this work, results related to the temperature influence on the homo-epitaxial growth process of 3C-SiC is presented. The seed for the epitaxial layer was obtained by an innovative technique based on silicon melting: after the first step of the hetero-epitaxial growth process of 3C-SiC on a Si substrate, Si melts, and the remaining freestanding SiC layer was used as a seed layer for the homo-epitaxial growth. Different morphological analyses indicate that the growth temperature and the growth rate play a fundamental role in the stacking faults density. In details, X-ray diffraction and micro-Raman analysis show the strict relationship between growth temperature, crystal quality, and doping incorporation in the homo-epitaxial chemical vapor deposition CVD growth process of a 3C-SiC wafer. Furthermore, photoluminescence spectra show a considerable reduction of point defects during homo-epitaxy at high temperatures.

## 1. Introduction

Silicon carbide is a promising new semiconductor material for high power, high frequency, and harsh environment device applications. For Metal Oxide Semiconductor Field Effect Transistor (MOSFET) application in the field of power-switching devices, 3C-SiC could be the best choice due to the minor electron trapping effect by the near-interface-traps density close to the oxide/semiconductor interface. This phenomenon can be avoided due to the smaller band-gap of the 3C-SiC compared with the other SiC polytypes [1,2].

3C-SiC (cubic structure) is the only one with cubic symmetry with notably advanced electrical properties, such as high electron mobility and high saturated electron velocity. Due to the cubic structures, it can be grown at much lower temperatures than other polytypes by a Chemical Vapor Deposition (CVD) reactor and directly on a silicon substrate. It allows the use of large-diameter Si substrates (greater than six inches), which greatly reduces fabrication costs.

The main issue in developing 3C-SiC devices is the lack of available material [3]. Bulk crystal with good crystal quality is not yet commercially available. This limitation is probably due to the fact that almost of the studies related to 3C-SiC growth were performed using Si as a substrate for the epitaxial process. Actually, the hetero-epitaxial system is limited by the high lattice mismatch between SiC and Si (20%), and the difference in thermal expansion coefficient that generates a very high density of defects during the grown of the layers and the cooldown, respectively [4,5]. In the last decades, many efforts have been devoted to searching for technologically feasible growth techniques to overcome such limitation. 

After the initial attempt by Nishino et al. [6], when he proposed a multi-step CVD process able to ensure an improvement in the crystal quality of the 3C-SiC epilayers, today, thick 3C-SiC films have been commonly grown on carbonized Si substrates by chemical vapor deposition. Important progress has been demonstrated using patterned (undulant) silicon substrates [7] followed by the switch-back epitaxy process. This technique seems to be promising, but the wafer bending issue remains unsolved. Another way is to use a silicon substrate with inverted silicon pyramids (ISPs) [8]. Using ISP substrate, a reduction of stacking faults (SFs) is observed for thin layers because this substrate allows for the probability of SFs annihilation, but the substrate manufacturing is not easy. Different approaches are being developed to find a convincing alternative to the 3C-SiC/Si heteroepitaxial system. In order to grow 3C-SiC bulk material of four and six inches, a new epitaxial reactor chamber has been designed and tested. In this work, the main idea is to grow a heteroepitaxial layer as seed, melt the silicon substrate, and continue the growth at a high temperature. In this way, it will be possible to grow a bulk substrate of 3C-SiC with a low density of SFs and low wafer bow. In fact, the bow can be strongly decreased, because by removing silicon, one of the main components of the stress due to the different thermal expansion coefficient between the two materials is completely eliminated. The removal of silicon also gives the possibility of a large increase in the growth temperature and in the growth rate, and then thicker wafers and better material can be grown [3].

## 2. Experimental

The growth of the 3C-SiC seed was performed with an epitaxial chemical vapor deposition (CVD) process, in a horizontal hot-wall reactor (LPE M-10, Catania, Italy) on a Si (100) substrate. Trichlorosilane (SiHCl_3_ or TCS), ethylene (C_2_H_4_), and hydrogen (H_2_) were used as silicon and carbon precursors and a gas carrier, respectively. The process was carried out in a low-pressure regime (100 mbar) at a temperature of 1400 °C (very close to the silicon melting temperature). After growing the buffer layer [9], the growth starts at 3 μm/h, then increases to 6 μm/h, and finally further increases to 30 μm/h. With this process, a thick layer of about 90 μm was grown. Subsequently, the temperature was increased above the melting point of silicon, and the Si substrate was completely melted inside the CVD reactor. The remaining freestanding SiC layer was used as a seed layer for subsequent homo-epitaxial growth. The processes were realized in a low-pressure regime at different temperatures in the range between 1600 and 1700 °C. A growth rate of 60 μm/h for two hours was used to increase the substrate thickness and 30 μm/h for the last 1 h to further improve the quality of the material, as previously reported [3]. While the first 20 microns of the last step were highly doped, the final 10 microns were low doped for device realization. Nitrogen was used as the doping species for n+ and n type layer formation. After the homo-epitaxial deposition, the total thickness of 3C-SiC samples was about 200 μm (observed by SEM analysis). 

Micro-Raman (Horiba JobinYvon, Longjumeau, France) and X-ray Diffraction (XRD) analyses were adopted to investigate the crystalline quality and structural order of the epitaxial layer. 

Micro-Raman analyses were collected using an HR800 integrated system (Horiba JobinYvon, Longjumeau, France) by Horiba JobinYvon in a back-scattering configuration. The excitation source was supplied by a He-Cd laser with a wavelength of 325 nm and was focalized on the sample by a 40X objective. By micro-Raman, the doping concentration of the epitaxial layer was also measured.

The crystal structure was investigated by high-resolution X-ray diffraction analysis (Bruker AXS, Karlsruhe, Germany). The measurement was performed using 2θ-ω scan and rocking curves (rc) on the (002) 3C-SiC plane. The diffractometer was equipped with a Eulerian cradle using a Cu–K source at a wavelength of 1.54 °A and operating at 40 kV and 40 mA. High-resolution optics, consisting of a two-bounced Ge022 monochromator and a very narrow variable slit system before the zero-dimensional detector, was adopted in order to obtain accurate data about the structural information of the samples.

Molten KOH etching methods were adopted for the SF evaluation. The etching in potassium hydroxide (KOH) was performed at 500 °C for 3 min. The densities were calculated based on the observation under fully automated (X, Y, and Z) optical microscopy. With powerful software for the image analysis coupled with a high-resolution microscope, a detailed dislocation count and classification were obtained.

The PL measurements were performed using a Horiba FLuorolog spectrofluorometer (Horiba JobinYvon, Longjumeau, France). The excitation wavelength selected was 370 nm in all experiments, and the emitted light was detected with a Peltier-cooled InGaAs array.

## 3. Results and Discussion

**Figure 1:**
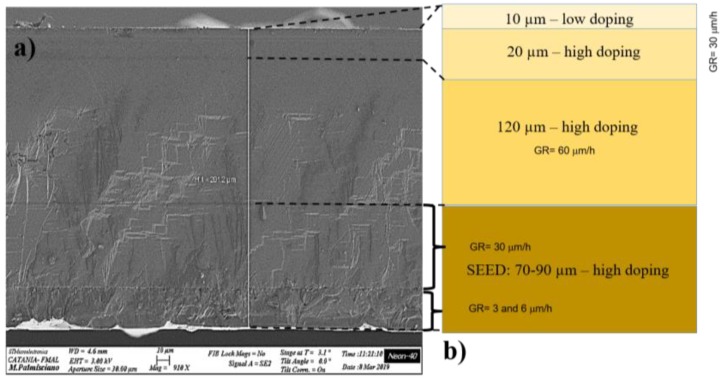
(**a**) Cross section SEM image of the 3C-SiC wafer (about 200 μm); (**b**) schematic representation of the structure: heteroepitaxial seed and homo-epitaxial layer. Layers have a different doping concentration and growth rate.

The first structural investigation of the entire 200 µm-thick film was performed by SEM in cross-section, where the 3C-SiC film (seed and homo-epitaxial layer) is observed (Figure 1). Figure 1a shows different layers within the film: starting from the bottom, first, the seed of 90 µm, and next, the homo-epitaxial layer grown after the fusion of Si. The seed shows different layers with different growth rates, with more defects close to the old silicon interface. The seed shows the decreasing of defects with the increasing growth rate. The second step is made up of the homo-process that reveals a lower thickness value (around 120 µm) compared to the predicted one (150 µm of Figure 1b). From the figure, the change of the doping concentration is also visible close to the film surface (10 µm-thick layer with low doping concentration close to the surface).

**Figure 2:**
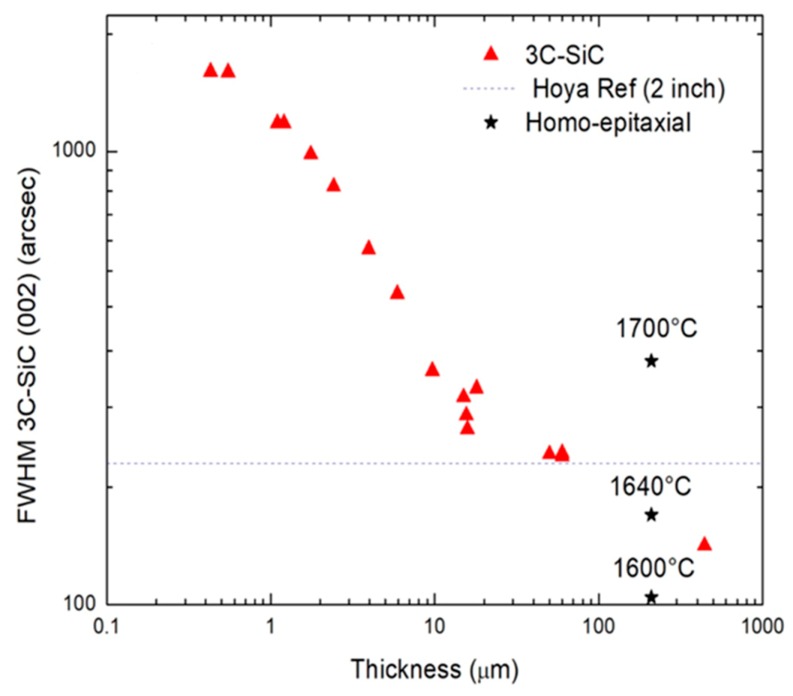
The full width of half-maximum (FWHM) of the X-ray Rocking curve as a function of film thickness for different values of temperature and compared with other reference samples [3].

The investigation of the temperature influence on the homo-epitaxial process was evaluated by X-ray diffraction analysis (Figure 2). The full width of half-maximum (FWHM) on the X-ray rocking curve of the 3C-SiC (002) peak is related to the crystal quality and defect density (decrease the FWHM, increase the crystal quality). Figure 2 shows the trend of FWHM as a function of film thickness for different sample growth, also in different conditions (all the samples with a low thickness value sourced from previous experiments [3]). It is well known that by increasing the film thickness, the quality of the material increases [10]. The first part of the curve (from 0 to about 20 μm of thickness) shows the 3C-SiC sample growth with the Si substrate. For such samples, the crystal quality is limited by the presence of silicon substrate that, starting from about 20 microns of thickness, introduces many cracks and extended defects during the cooling down after the growth [3]. The three points at 60 μm are the 3C-SiC samples without silicon substrate (after Si fusion) with a good crystal quality (around 200 arcsec), comparable with an old two-inch 3C-SiC wafer provided by Hoya corporation (dotted line). These samples are the substrates used as the template for the homo-epitaxial process of this experiment. The starred points (three at 200 microns) are the samples that grew using the new melting technique described in the present work. They were grown at three different temperatures (1600 °C, 1640 °C, and 1700 °C). The graph clearly shows that the sample grown at 1600 °C is significantly better than the other two. Indeed, it has an FWHM value of around 100 arcsec that is very promising, also compared to a thicker sample (about 400 microns) grown by sublimation epitaxy (PVT reactor) at high temperature [11].

**Figure 3:**
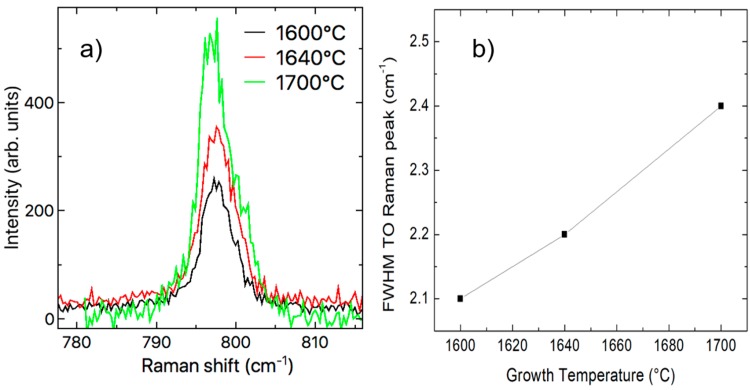
(**a**) The intensity of the Transversal Optical (TO) Raman mode peak for the different growth temperatures studied; (**b**) FWHM of the TO curve as a function of the growth temperature.

In the 3C-SiC, the micro-Raman signal of the transversal optical (TO) mode in our experimental conditions (back scattering configuration and (001) oriented samples) should be forbidden [12] due to selection rules. In the “perfect” cubic structure (001)-oriented, the TO Raman peak is not allowed, for this reason, the TO peak could be considered as a marker of the quality of the material. Nevertheless, the defects and stress produce a loss of periodicity, reduction of crystal symmetry, and topological disorder of crystals. Therefore, they induce a breakdown of the selection rules and the appearance of such a peak in the experimental spectra. Figure 3 shows the TO intensity peak for samples grown at different growth temperatures. The decreasing signal intensity means a decreasing of crystal “disorder”. Lower intensity was recorded for samples grown at 1600 °C. Moreover, the FWHM of the TO mode is related to the crystal quality of the film. In Figure 3b the FWHM of the TO mode (reported in Figure 3a) as a function of growth temperature is reported. The graph clearly shows that the quality of the material improves when lowering the growth temperature. These results confirm the XRD result reported in Figure 2.

**Figure 4:**
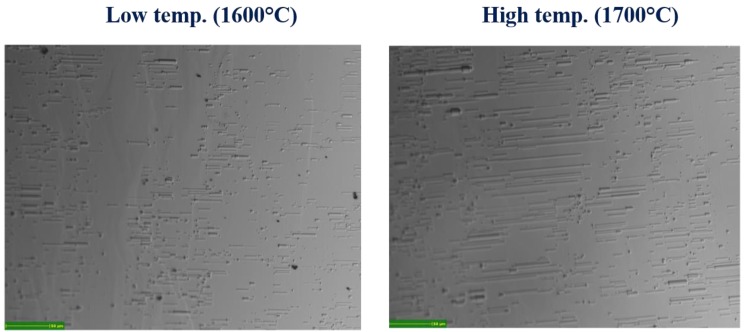
Optical microscope image of the sample growth at 1600 °C (**left**) and 1700 °C (**right**) after the molten KOH etching at 500 °C for 3 min. The figure shows the shape of the stacking faults after etching.

The Stacking Faults (SFs) density on the surface was investigated by etching epilayer in molten KOH and observed by a fully automated optical microscope. Image analysis software allows to count and classify SFs. Figure 4 shows the optical microscope image of the sample growth at 1600 °C (left) and 1700 °C (right). The figure shows the shape of the stacking faults after the etching (line-like). By comparing the figures, we can observe that the total amount of SFs is lower for the low-temperature sample and the low temperature also shows the lower SFs length. At high temperatures, the number of short SFs decreased and, at the same time, the SFs became longer. The increased amount of SFs at high growth temperatures is the reason why both the XRD and micro-Raman defect evaluation analysis shows better crystal quality at lower temperatures. 

**Figure 5:**
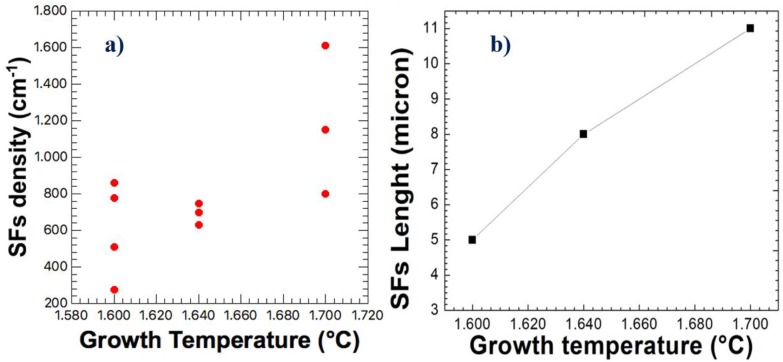
(**a**) Stacking Faults density as a function of the growth temperature obtained by optical observation of the 3C-SiC layer; (**b**) Stacking Faults length as a function of growth temperature. The data were obtained after the molten KOH etching of the 3C-SiC wafer at 500 °C.

In Figure 5, the results of the SFs analysis, based on the previous images, on the 3C-SiC film as a function of deposition temperature, are reported. From the SFs data analysis, an increase of both the SF density (see Figure 5a) and SF length (see Figure 5b) was observed with increasing temperature. 

In a previous paper [13], it was observed that two kinds of SFs could be distinguished in 4H-SiC. A kind of stacking faults can be “closed” by using high temperature and low growth rate, while the other kind can be closed by using a low temperature and high growth rate. This kind of study has not been performed in the case of 3C-SiC growth, but from these measurements, it seems that the low temperature can help to close the SFs observed in 3C-SiC [13].

Probably for kinetic reasons, the combination of 30 μm/h with 1600 °C allows for a reduction in SFs density with respect to the 1700 °C growth. At a lower temperature, the kinetics of the system produces growth of the defect-free material (without SF) surrounding the SF and allows the closure of the SF on itself. For this reason, at a lower temperature, we observe a lower “long SF” density and the closure of short ones. Probably, by increasing the growth time, in these conditions, we could observe a further decrease of the SFs density.

**Figure 6:**
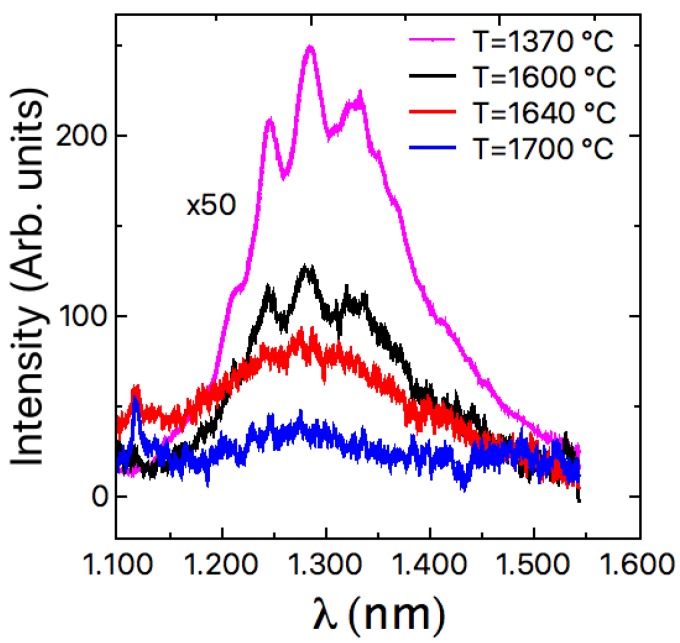
Infrared PL spectra for different grown temperatures. For the seed sample grown at a lower temperature (1370 °C), the point defects peak is 50 times reduced in intensity when shown in the same scale.

In Figure 6, the PL spectra of the different samples in the wavelength range 1100–1600 nm, are reported. The PL spectra in the usual range between 450 and 900 nm do not show any particular peak structure with the exception of the band-band peak at 540 nm. As reported in previous papers, the SFs in 3C-SiC do not produce a PL peak [14], as in the case of 4H-SiC, because they do not introduce levels inside the band-gap [15]. Then, the emission in this wavelength range of Figure 6 close to the mid-gap of 3C-SiC, is attributed to points defects, as reported in a previous paper where 3C-SiC had been implanted with different ions and annealed at different temperatures [16]. It can be observed that the signal at 1300 nm is extremely high in the seed layer (standard heteroepitaxial growth process at a low temperature, 1370 °C, with silicon substrate) without the homo-epitaxial layer on top. The PL emission decreases, increasing the growth temperature at a fixed growth rate. A similar behavior that confirms what was observed in Figure 6, has been reported previously also by Kinetic Monte Carlo Simulations (KMCS), and by Schottky diodes leakage current distributions in a previous paper on 4H-SiC [17]. It has been observed that the point defects density depends essentially on the growth temperature and the growth rate. Low growth rate and high temperature decrease the point defects density, while high growth rate and low temperature increase the point defects density.

Micro-Raman of Longitudinal Optical mode (LO) was also performed. From these measurements, it is possible to determine the doping concentration of both the 10 μm-thick low doped region and of the high doped region of the substrate. The obtained results are reported in Table 1. For a fixed value of N, the doping concentration of the fixed layer is 4 × 10^17^/cm^3^ for all the temperatures. The other LO peak reveals that the doping incorporation change as a function of the temperature, increasing with temperature.

The interaction between electron collective excitations with the longitudinal optical phonon leads to LO phonon-plasmon coupled (LOPC) mode whose Raman line shape is particularly sensitive to ionized dopant concentration in inspected material. By fitting Raman LO peak with its intensity equation,

(*ω*) = *S*(*ω*)*Im*{−1/*ε*(*ω*), (1)

where ω is the Raman shift, *S* is a proportionality constant, *ε* is the dielectric function, and A(*ω*) is given in reference [18], we were able to estimate sample carrier density. In fact, by entering the nominal 3C-SiC TO and LO vibrational frequency, namely $\omega_{T}$ and $\omega_{L}$, in the dielectric function expression

(2) $$\varepsilon \left(\omega \right)=\varepsilon_{\infty }\{1+[\omega_{L}^{2}-\omega_{T}^{2}/(\omega_{T}^{2}-\omega^{2}-i\omega \Gamma )]\}-[\omega_{p}^{2}/\omega \left(\omega +i\gamma \right)],$$

where $\varepsilon_{\infty }$ is the infinite dielectric constant, Γ and γ are the phonon and electron damping factor, respectively, it is possible to extract the electron carrier density from the expression of plasma frequency

(3) $$\omega_{p}=\sqrt{4\pi ne^{2}/\varepsilon_{\infty }m},$$

where *m* is the electron effective mass. This technique provides a non-destructive immediate way for electrical sample characterization [19].

## 4. Summary

In conclusion, the quality of homo-epitaxial 3C-SiC growth with an innovative technique is presented. This new approach consists of the heteroepitaxial growth of 3C-SiC on Si with, subsequent melting of the Si wafer and the homo-epitaxial growth of 3C-SiC. In this work, the quality of the homo-epitaxial grown 3C-SiC wafer is widely investigated. All the analysis related to the crystal structure reveals that SFs density increases, increasing the growth temperature from 1600 °C to 1700 °C. On the other hand, the density of point defects decreases, increasing the growth temperature, from 1370 °C (heteroepitaxial process with Si substrate) to the homo-epitaxial growth at 1700 °C. The doping concentration also increases at a high growth temperature.

## Figures and Tables

**Table 1:** Doping concentration.

Growth Temperature (°C)	Low Doping (cm^−3^)	High Doping (cm^−3^)
1600	4 × 10^17^	2.1 × 10^18^
1640	3.6 × 10^17^	3.3 × 10^18^
1700	4 × 10^17^	6.2 × 10^18^
